# Global proteomic analysis of the oocyst/sporozoite of *Toxoplasma gondii* reveals commitment to a host-independent lifestyle

**DOI:** 10.1186/1471-2164-14-183

**Published:** 2013-03-15

**Authors:** Alessia Possenti, Federica Fratini, Luca Fantozzi, Edoardo Pozio, Jitender P Dubey, Marta Ponzi, Elisabetta Pizzi, Furio Spano

**Affiliations:** 1 Department of Infectious, Parasitic and Immunomediated Diseases, Istituto Superiore di Sanità, Viale Regina Elena 299, Rome 00161, Italy; 2 United States Department of Agriculture, Agricultural Research Service, Animal Parasitic Diseases Laboratory, Animal and Natural Resources Institute, Beltsville, MD, 20705, USA; 3 Present address: Agenzia Regionale per la Protezione Ambientale del Lazio, Sezione di Rieti, Via Salaria per l'Aquila 8, Rieti, 02100, Italy

**Keywords:** *Toxoplasma gondii*, Oocyst, Sporozoite, Proteome, Metabolism, Energy production, Host cell invasion, Oocyst-specific proteins

## Abstract

**Background:**

Toxoplasmosis is caused by the apicomplexan parasite *Toxoplasma gondii* and can be acquired either congenitally or via the oral route. In the latter case, transmission is mediated by two distinct invasive stages, i.e., bradyzoites residing in tissue cysts or sporozoites contained in environmentally resistant oocysts shed by felids in their feces. The oocyst plays a central epidemiological role, yet this stage has been scarcely investigated at the molecular level and the knowledge of its expressed proteome is very limited.

**Results:**

Using one-dimensional gel electrophoresis coupled to liquid chromatography-linked tandem mass spectrometry, we analysed total or fractionated protein extracts of partially sporulated *T. gondii* oocysts, producing a dataset of 1304 non reduntant proteins (~18% of the total predicted proteome), ~59% of which were classified according to the MIPS functional catalogue database. Notably, the comparison of the oocyst dataset with the extensively covered proteome of *T. gondii* tachyzoite, the invasive stage responsible for the clinical signs of toxoplasmosis, identified 154 putative oocyst/sporozoite-specific proteins, some of which were validated by Western blot. The analysis of this protein subset showed that, compared to tachyzoites, oocysts have a greater capability of de novo amino acid biosynthesis and are well equipped to fuel the Krebs cycle with the acetyl-CoA generated through fatty acid β-oxidation and the degradation of branched amino acids.

**Conclusions:**

The study reported herein significantly expanded our knowledge of the proteome expressed by the oocyst/sporozoite of *T. gondii,* shedding light on a stage-specifc subset of proteins whose functional profile is consistent with the adaptation of *T. gondii* oocysts to the nutrient-poor and stressing extracellular environment.

## Background

The obligate intracellular parasite *Toxoplasma gondii* is a protozoan of the Phylum Apicomplexa highly prevalent in humans and animals worldwide [1]. Its clinical relevance is mainly due to potential congenital transmission to the fetus by seronegative women, causing intrauterine death or severe sequelae in the newborn [2] and adult life [3]. Furthermore, reactivation of latent infections in immunocompromised subjects can lead to life-threatening encephalitis. The complex life cycle of *T. gondii* includes three infectious stages, the tachyzoite, the bradyzoite and the sporozoite [4]. The rapidly dividing tachyzoite is responsible for the clinical signs of toxoplasmosis, as it disseminates the infection to virtually all organs and tissues of the host and can reach the fetus transplacentally. However, the major role in disease transmission is played by the oral ingestion of either bradyzoites encysted in the tissues of chronically infected hosts or sporozoites contained in the oocyst. This highly resistant environmental stage is the result of sexual reproduction, which occurs in the intestinal epithelium of cats and virtually all species of felids and culminates in the fecal shedding of unsporulated oocysts [5]. In the external environment, oocysts undergo sporogony [6] and develop two ellipsoidal sporocysts containing four infectious sporozoites each, which can survive in moist soil or water for months to years [1].

Despite its crucial epidemiological role, the sporozoite is the less characterized at the molecular level among the infectious stages of *T. gondii*, mostly due to the difficulty in producing and handling oocysts. The existence of oocyst/sporozoite-specific proteins was first documented nearly thirty years ago by serological and biochemical methods [7,8], yet no stage-specific protein was identified until 2004, when Radke and colleagues characterized the major sporozoite surface antigen SRS28 (SporoSAG) [9]. Subsequently, we reported on the cloning and immunolocalization of the cysteine-rich oocyst wall proteins TgOWP1, TgOWP2 and TgOWP3 [10], whereas Hill et al. [11] employed a proteomic-based approach to demonstrate that sporozoites specifically express the immunogenic embryogenesis-related protein (TgERP). In recent years, several groups applied different high-throughput proteomic techniques to the study of the tachyzoite of *T. gondii*. The analysis of whole [12,13] or enriched [14-21] protein extracts provided an extensive coverage of the protein complement expressed by this parasite stage, with >5000 distinct proteins identified, which account for ~73% of the global predicted proteome. Following large-scale proteomic analyses of the oocyst stage of two other apicomplexans, i.e., *Cryptosporidium parvum*[22,23] and *Eimeria tenella*[24], very recently Fritz and co-workers published a proteomic study of fully sporulated oocysts of the *T. gondii* strain M4 (genotype II) [25]. Using one-dimensional (1-D) gel electrophoresis coupled to liquid chromatography (LC)-linked tandem mass spectrometry (MS/MS), the authors analysed protein extracts from either oocyst wall or sporocyst/sporozoite fractions and produced a first proteomic chart of 1031 individual proteins, including small subsets of molecules possibly implicated in oocyst environmental resistance and moving junction formation.

In the present work, we report on the proteomic analysis of partially sporulated oocysts of *T. gondii* belonging to the genotype III strain VEG. The identification of 1304 non redundant proteins increased significantly the coverage of the oocyst/sporozoite proteome and allowed to expand our knowledge of developmentally regulated protein expression in *T. gondii*, highlighting differences between the repertoires of metabolic enzymes expressed by the oocyst/sporozoite and tachyzoite stage.

## Results

### Analysis of the oocyst/sporozoite proteome of *T. gondii* by one-dimensional electrophoresis gel LC MS/MS

To shed light on the expressed proteome of the oocyst/sporozoite of *Toxoplasma gondii*, total proteins were extracted from partially sporulated oocysts of the type III strain VEG [1] and analysed by one-dimensional gel electrophoresis LC-MS/MS in three independent experiments (Additional file 1). The oocyst batch used throughout the study consisted of approximately 60% fully sporulated and 40% non sporulated oocysts, as estimated by light microscopy observation.

**Figure 1:**
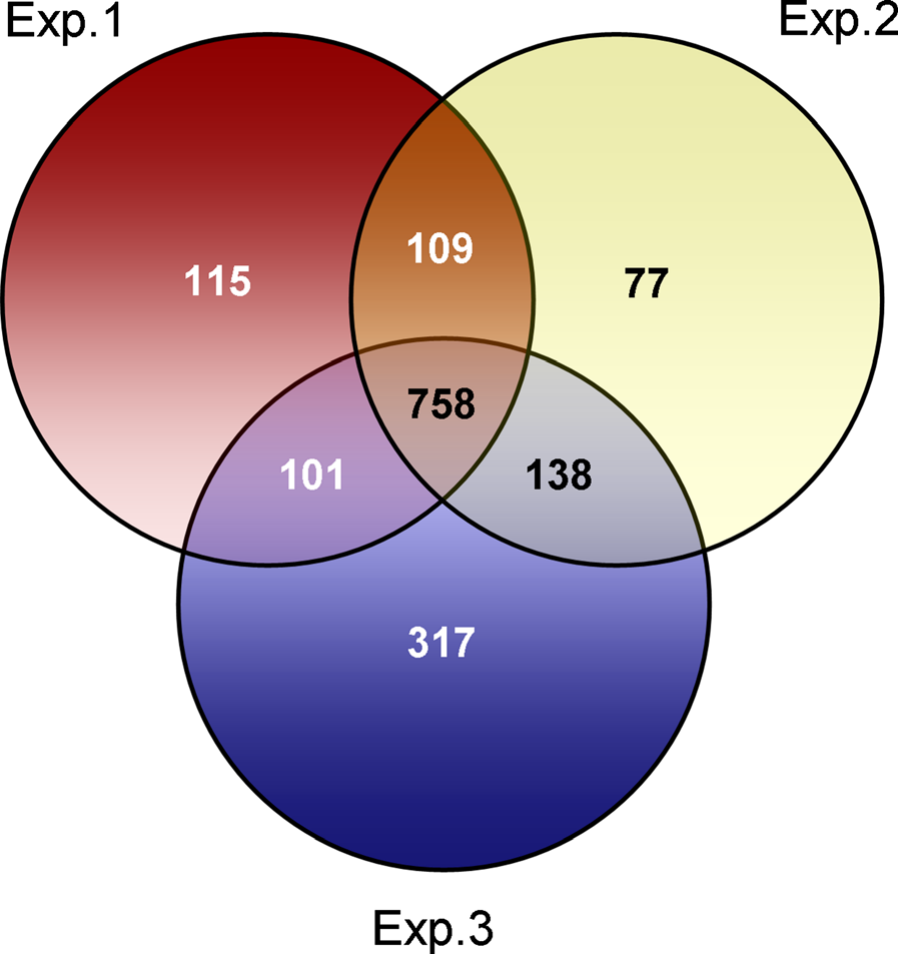
**Comparison between independent proteomic experiments. **The Venn diagram shows the number of unique and shared oocyst/sporozoite proteins identified by LC-MS/MS in three independent biological replicates. Experiments 1, 2 and 3 allowed to identify 1083, 1082 and 1314 *T. gondii *proteins, respectively, with overlaps among different experiments comprised between 65% and 83%.

Peptide sequences generated from raw mass spectra were initially matched against the release 5.1 of ToxoDB (http://www.toxodb.org/), yielding 6114 individual protein identifications. The entire dataset was successively updated on the release 7.1 of the database. After removal of redundancies, mostly due to multiple identifications of the same gene in different parasite reference strains, experiments 1, 2 and 3 allowed the identification of 1083, 1082 and 1314 *T. gondii* proteins, respectively (Figure 1), with overlaps between different experiments of between 65% and 83%. Approximately 47% of the proteins (758) were identified in all experiments, whereas varying numbers were detected uniquely in experiment 1 (115), 2 (77) or 3 (317) (Figure 1). The three independent analyses yielded a combined dataset of 1615 non redundant oocyst/sporozoite proteins (Additional file 2) accounting for ~22% of the ToxoDB predicted proteome, which, according to a recent estimate, consists of ~7300 proteins [26]*.* According to ToxoDB annotations, 25% of the 1615 proteins identified in this study contain a signal peptide and 18% possess at least one transmembrane domain, two structural features occurring in very similar proportions in the predicted proteome of *T. gondii,* i.e., 21% and 18%, respectively. Approximately 38% of the oocyst/sporozoite identifications correspond to “hypothetical” proteins, which otherwise account for nearly 60% of the entire predicted proteome.

### Functional analysis of the protein dataset

**Figure 2:**
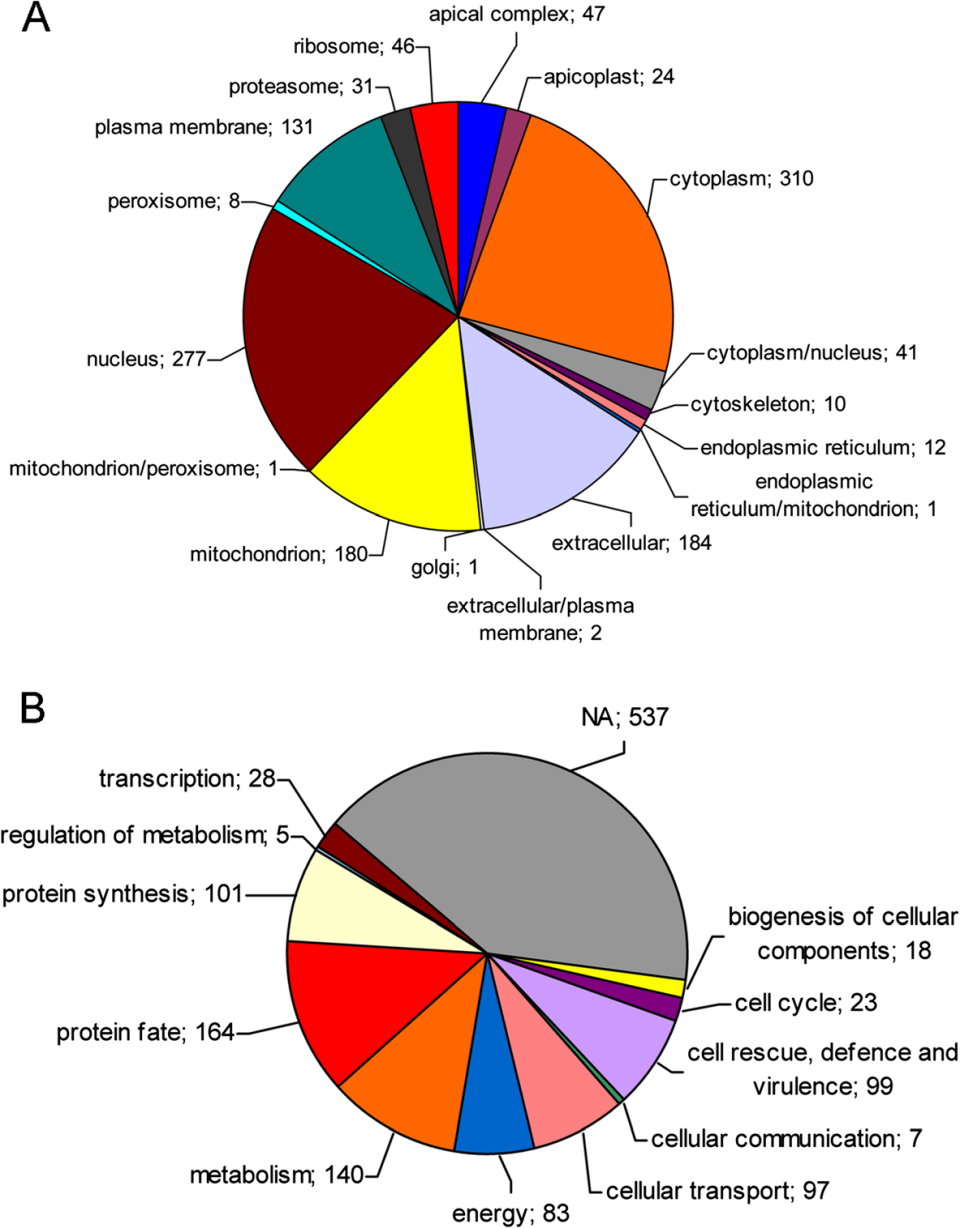
**Predicted subcellular localization and functional classification of oocyst/sporozoite proteins. **(**A**) The proteins identified were assigned a subcellular localization combining the results of the general predictor WoLF PSORT with those of the programs PlaMit and PATS for the identification of mitochondrial and apicoplast targeting signals, respectively. (**B**) Proteins were classified into functional categories according to the MIPS functional catalogue database. Assignments were based on GO biological process annotations provided by ToxoDB, on annotations of the KEGG Pathway database and on independent BLAST homology and literature searches. NA, not assigned. The number of proteins in each class is indicated.

Of 1615 proteins, 311 (19%) were identified with a single peptide in one experiment and were excluded from functional analyses. The remaining 1304 proteins were assigned a subcellular localization (Figure 2A) combining the Gene Ontology (GO) cellular component predictions available in ToxoDB, with the results produced by the general subcellular predictor WoLF PSORT. Putative mitochondrial and apicoplast localizations were further assessed using the PlasMit and PATS predictors, respectively. Proteins with cytoplasmic (24%), nuclear (21%) and mitochondrial (14%) localizations were highly represented, whereas nearly one-third of the molecules identified were associated with parasite’s secretory routes, including those assigned to extracellular locations (14%), the plasmamembrane (10%) and the apical complex (4%). The latter category included 47 micronemal and rhoptry proteins, many of which have been thoroughly characterized in recent years.

Of 1304 proteins, 767 (~59%) were assigned a functional category according to the MIPS functional catalogue database (FunCatDB) (Figure 2B). The vast majority of these proteins were classified using the GO biological process annotations of ToxoDB, which cover only 25% of the predicted proteome. A small fraction of the proteins involved in metabolic processes was identified using the KEGG Pathway database (http://www.genome.jp/kegg/), whereas approximately 80 molecules were assigned to a MIPS category based on sequence similarities with functionally characterized *T. gondii* paralogs, independent BLAST homology searches or exploiting information on subcellular localization contained in recent literature. The most numerous functional categories included proteins involved in protein fate (164), metabolism (140), protein synthesis (101), cell rescue, defence and virulence (99) and cellular transport (97).

The comparison of the VEG oocyst proteins with the expressed tachyzoite proteome provided a global, yet not exhaustive, view of the different protein repertoires ex-pected to play a role in host cell invasion by *T. gondii* sporozoites. Our dataset comprises 9 proteins belonging to the SRS superfamily [27], which consists of >100 GPI-anchored surface ligands related to SRS29B (SAG1), an immunodominant protein highly expressed on tachyzoites. We also detected 16 proteins (MICs) localized to the micronemes, the apical secretory organelles involved in parasite motility and/or invasion, and several invasion-related molecules residing in a second type of apical organelle, i.e., the club-shaped rhoptry [28]. These included 11 rhoptry neck proteins (RONs), which are essential constituents of the moving junction [29,30], and 28 rhoptry bulb proteins (ROPs), which are secreted into the nascent parasitophorous vacuole (PV) or injected into the host cell cytoplasm, thus participating in the modification of the PV membrane or in the subversion of host cell transcription. The oocyst dataset included also 8 proteins localized to the dense granules (GRAs), which release their content into the vacuolar space in the late phases of invasion [31].

### Correlation between proteomic and transcriptional data

**Figure 3:**
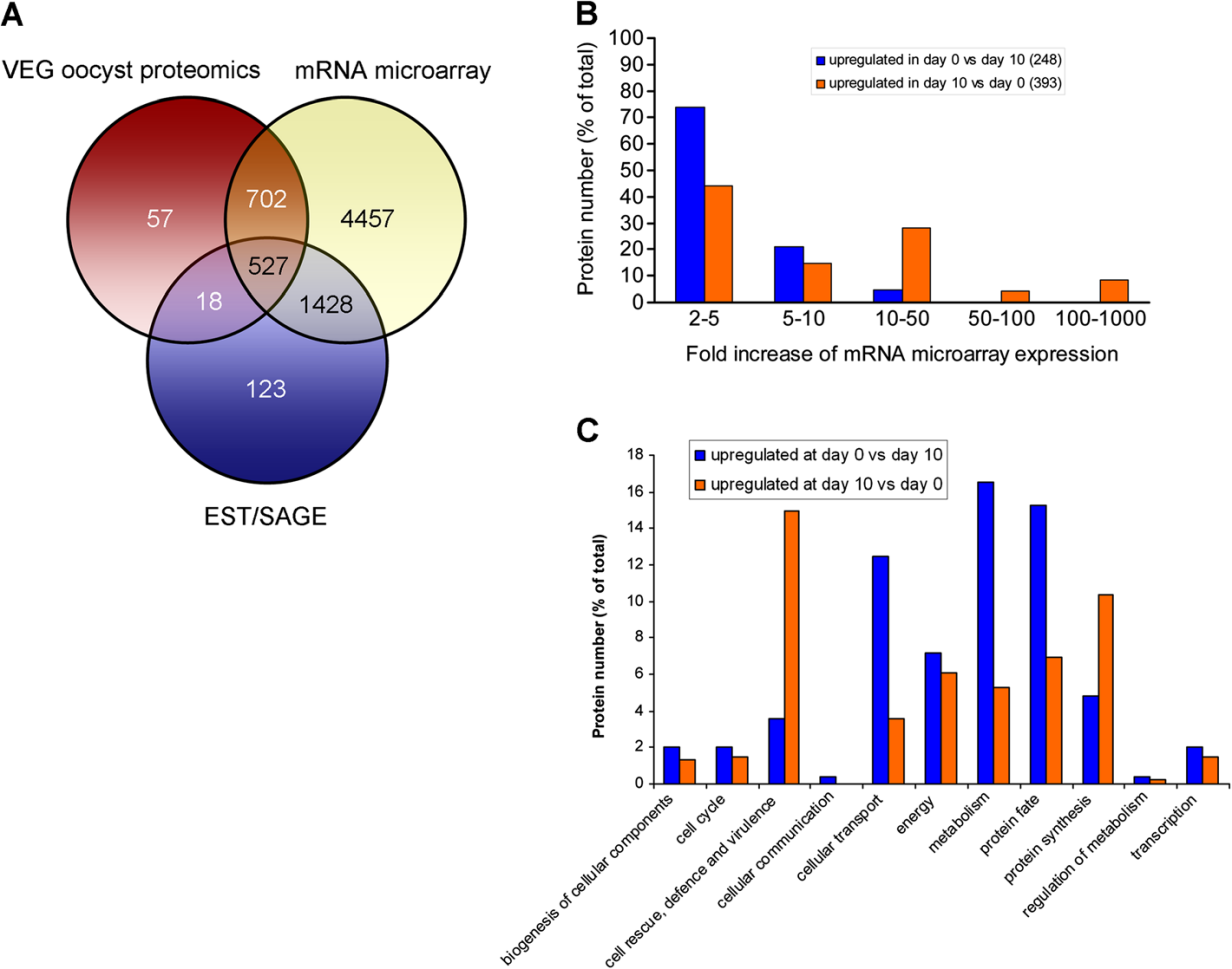
**Comparison between proteomic and transcriptional data of *T. gondii *oocysts. **(**A**) Venn diagram showing the overlap between 1304 oocyst proteins identified in this study and different types of transcriptional data incorporated in ToxoDB 7.1. Microarray data refer to mRNA expression of 7114 *T. gondii *genes in sporulated oocysts of strain M4 [34]. ESTs from sporulated and unsporulated oocyst (1167) and 1269 sporozoite SAGE tags (≥2 counts, 1 occurrence/genome, sense) [33] of strain VEG were combined with each other to eliminate redundancies prior to comparison with the proteomics and microarray datasets. (**B**) The histogram shows the distribution of 1304 *T. gondii *genes proteomically identified in this study among various classes of mRNA microarray expression [34]. Numeric ranges indicate the fold increase of mRNA levels in usporulated (day 0) versus sporulated (day 10) oocysts and vice versa. Were included in the analysis 248 and 393 genes showing a transcriptional fold increase ≥2 at day 0 or day 10, respectively. (**C**) Classification into MIPS functional categories of the proteins encoded by genes exhibiting a relative transcriptional upregulation ≥2 in day 0 or day 10 oocysts.

To gain insight into the relationship between protein and mRNA expression [32], we matched the proteomic data generated in the present study with oocyst/sporozoite-specific transcriptional data available in ToxoDB 7.1 (Figure 3A). These included 1167 non redundant ESTs from partially (999) or fully (250) sporulated oocysts, 1269 non redundant sporozoite SAGE tags [33] and mRNA microarray data on 7114 *T. gondii* genes relative to M4 oocysts at various sporulation stages [34]. Using ToxoDB bioinformatic tools, we analysed the M4 microarray data to identify genes upregulated in day 0 (unsporulated) versus day 10 (fully sporulated) oocysts and vice versa. Excluding genes with a transcriptional fold increment <2, we found that 248 (19%) and 393 (30%) proteins out of 1304 were encoded by genes showing transcriptional upregulation in day 0 or day 10 oocysts, respectively (Figure 3B). At day 0, only 4.8% of upregulated genes exhibited a rise of mRNA levels greater than 10-fold, with a maximum increment of 18.6-fold, whereas the vast majority (74%) showed a transcriptional increase of only 2- to 5-fold. This unequal distribution may reflect the fact that a significant fraction of these genes have a transcriptional peak in developmental stages preceeding the unsporulated oocyst, e.g., the macrogamete or the early zygote. Quite distinctly, ~41% of the genes upregulated in fully sporulated oocysts showed trancriptional increments higher than 10-fold, with 33 genes exhibiting fold increase values >100, e.g., the dense granule proteins GRA7 (933) and GRA1 (810), the microneme protein MIC11 (443) and the surface antigen SRS57 (367).

Based on the assignment to MIPS functional categories, the proteins encoded by genes upregulated in day 0 versus day 10 oocysts showed a higher relative abundance of molecules implicated in metabolism (16.5% versus 5.3%), cellular transport (12.5% versus 3.6%) and protein fate (15.3% versus 6.9%) (Figure 3C). On the other hand, genes upregulated at day 10 encoded higher proportions of molecules involved in protein synthesis (10.4% versus 4.8%) and in cell rescue, defence and virulence (15.0% versus 3.6%), with the latter group consisting mainly of proteins located in secretory organelles [35], i.e., the rhoptries (39), the micronemes (16) and the dense granules (8).

### Putative oocyst/sporozoite-specific proteins (POSPs)

**Figure 4:**
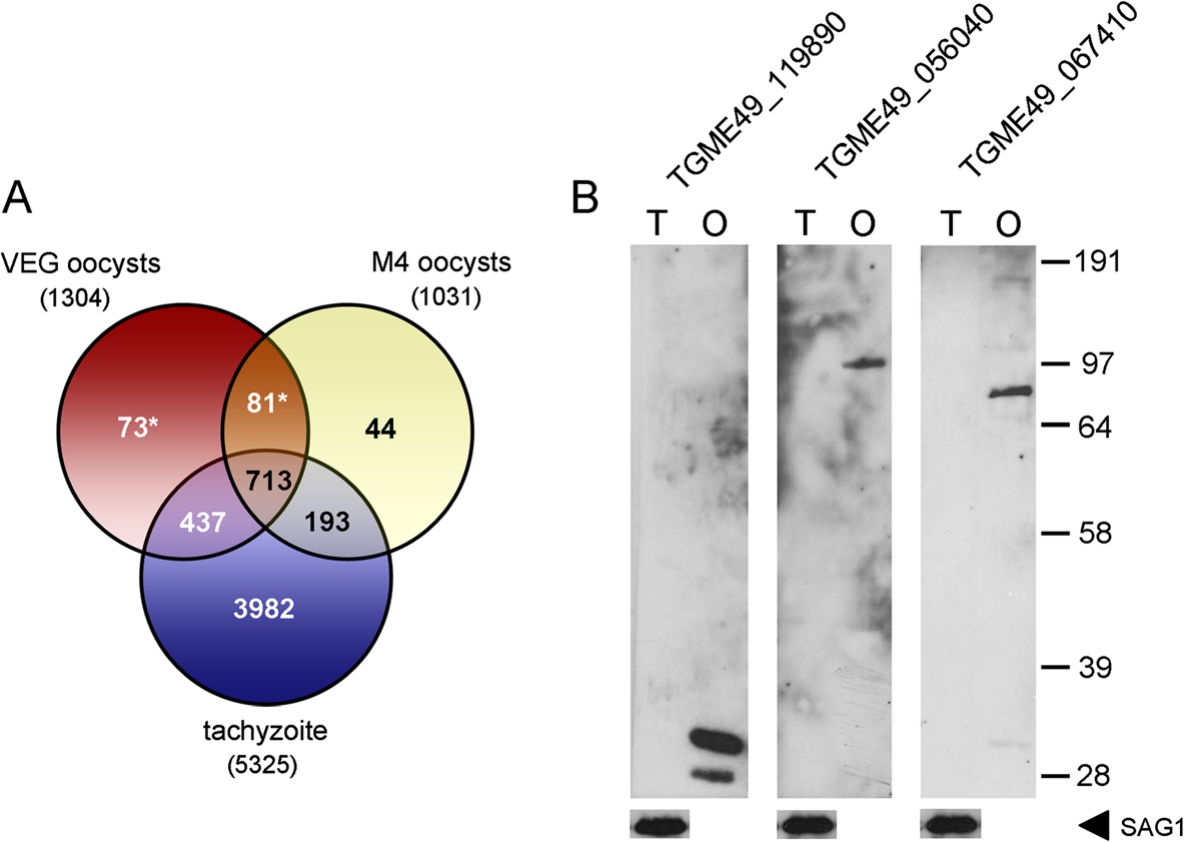
**Identification and validation of putative oocyst/sporozoite-specific proteins (POSPs).** (**A**) Comparison of the oocyst proteins identified in the present study with the expressed proteome of the tachyzoite and a dataset of proteomically identified proteins of sporulated M4 oocysts [25]. Numbers in brackets indicate the complexity of each dataset. The Venn diagram shows the presence in the VEG oocyst dataset of 154 proteins (POSPs), indicated by the asterisk, lacking proteomic evidence in tachyzoites. (**B**) Western blot analysis of total protein extracts from partially sporulated oocysts and tachyzoites of the VEG strain. The three panels show the reactivity of mouse polyclonal antibodies raised against three members of the POSP subset, i.e., the tyrosine-rich hypotetical protein TGME49_119890 and the LCCL domain-containing proteins TGME49_056040 and TGME49_067410. As quality and loading control, the tachyzoite lanes were also probed with an anti-SAG1 monoclonal antibody (arrowhead). O, oocyst; T, tachyzoite.

During the preparation of this manuscript, a considerable number of novel proteomic identifications was added to the release 7.1 of ToxoDB, including phosphoproteomic and total proteomic data on intracellular and extracellular tachyzoites [18,19] and the results of a global analysis of oocyst proteins fractionated into wall and sporocyst/sporozoite components [25]. Despite a marked cross-contamination of the analysed fractions, the latter approach led to the identification of 1031 proteins, 794 of which (77%) are represented in our dataset (Figure 4A). In addition, the proteins identified in the present study were filtered against the expressed tachyzoite proteome, which consisted of 5325 proteins resulting from the combination of global and subcellular proteomic data deposited in ToxoDB by various laboratories. As shown in Figure 4A, the oocyst/sporozoite dataset showed an overlap of ~88% with that of the tachyzoite and contained 154 proteins devoid of proteomic evidence in this stage. Given the numerous proteomic studies carried out on the tachyzoite and the consequent extensive coverage of its expressed proteome, these 154 unique proteins were likely to reflect developmentally regulated expression and were referred to as putative oocyst/sporozoite-specific proteins (POSPs) (Table 1). The stage specificity of three POSPs was tested by western blot using polyclonal antibodies raised against the LCCL domain-containing proteins TGME49_056040 and TGME49_067410 and the tyrosine-rich hypothetical protein TGME49_119890. As shown in Figure 4B, on protein lysates of partially sporulated VEG oocysts all antibodies yielded specific banding patterns compatible with the predicted molecular masses of the selected proteins, whereas no reactivity was observed on total tachyzoite extracts of the same parasite strain.

**Table 1:** List of putative oocyst/sporozoite-specific proteins (POSPs) identified in this study

**Gene ID**	**Protein name**	**EC number**	**Biological process/molecular function**	^**a**^**Fold change of oocyst vs tachyzoite mRNA levels**			**Protein probability**
				day 0	day 4	day 10	
***energy***							
TGME49_026300	oxidoreductase, putative	1.3.1.34	fatty acid β-oxidation	**260**	40	103	6.23E-11
TGME49_034570	peroxisomal multifunctional enzyme type 2, putative	1.1.1.35	fatty acid β-oxidation	**108**	12	17	2.22E-16
TGME49_042390	enoyl-CoA hydratase/isomerase family protein, putative	4.2.1.17	fatty acid β-oxidation	**21**	16	5	1.35E-12
TGME49_047500	acyl-coenzyme A oxidase, putative	1.3.3.36	fatty acid β-oxidation	**92**	37	49	6.76E-12
TGME49_066270	2-methylbutyryl-CoA dehydrogenase, putative	1.3.99.-	fatty acid β-oxidation	**7**	<2	<2	1.00E-30
TGME49_115480	acyl-CoA oxidase, putative	1.3.3.36	fatty acid β-oxidation	**183**	71	151	1.91E-10
TGME49_032580	acetyl-coenzyme A synthetase, putative	6.2.1.1	glycolysis/gluconeogenesis	**43**	5	12	1.00E-30
TGME49_089930	phosphoenolpyruvate carboxykinase, putative	4.1.1.49	glycolysis/gluconeogenesis	**30**	3	6	8.70E-07
TGME49_016090	tropomyosin 1 alpha chain, putative	-	regulation of actin mechanics	**247**	11	19	5.66E-06
TGME49_027050	pyruvate dehydrogenase kinase, putative	2.7.11.2	regulation of pyruvate dehydrogenase	3	**4**	3	2.16E-07
TGME49_071210	4-alpha-glucanotransferase, putative	2.4.1.25/3.2.1.33	starch and glucose metabolism	5	**254**	26	1.11E-15
TGME49_003110	citrate synthase, putative	2.3.3.1	TCA cycle	**35**	5	7	6.32E-09
***metabolism***							
TGME49_003340	alkyl sulfatase, putative	3.5.2.6	-	**8**	<2	2	2.10E-05
TGME49_003580	NAD-binding domain-containing protein	-	-	n.a.	n.a.	n.a.	4.73E-12
TGME49_038200	alpha/beta hydrolase fold domain containing protein	-	-	**27**	3	4	7.77E-15
TGME49_065860	nit protein 2, putative	-	-	**5**	<2	<2	2.74E-11
TGME49_069120	oxidoreductase, putative	1.1.1.100	-	**183**	61	125	4.44E-16
TGME49_069400	short chain dehydrogenase/reductase domain-containing protein	1.1.1.100	-	**11**	<2	<2	2.42E-07
TGME49_106450	short chain dehydrogenase family protein, putative	1.1.1.100	-	<2	**222**	82	6.97E-05
TGME49_111780	alcohol dehydrogenase class III, putative	1.1.1.284	-	**4**	<2	<2	4.61E-06
TGME49_003500	alanine dehydrogenase, putative	1.4.1.1	amino acid metabolism	6	**29**	4	1.11E-15
TGME49_005420	aspartate-semialdehyde dehydrogenase, putative	1.2.1.11	amino acid metabolism	**4**	<2	<2	2.44E-08
TGME49_016640	homoserine kinase, putative	2.7.1.39	amino acid metabolism	**74**	28	26	4.03E-12
TGME49_059180	cystathionine beta-synthase, putative	4.2.1.22	amino acid metabolism	n.a.	n.a.	n.a.	8.37E-13
TGME49_069110	ornithine aminotransferase, putative	2.6.1.13	amino acid metabolism	**394**	123	247	2.54E-12
TGME49_078910	O-acetylserine (thiol) lyase, putative	2.5.1.47	amino acid metabolism	**20**	3	5	2.39E-10
TGME49_112930	cystathionine beta-lyase, putative	4.4.1.1	amino acid metabolism	**23**	2	3	2.90E-10
TGME49_115260	alanine dehydrogenase, putative	1.4.1.1	amino acid metabolism	98	211	**331**	2.89E-14
TGME49_019230	acetoacetyl-CoA synthetase, putative	6.2.1.16	butanoate metabolism	<2	<2	<2	4.97E-10
TGME49_031900	acyl-CoA dehydrogenase, putative	1.3.99.7	fatty acid metabolism	31	**32**	21	6.51E-11
TGME49_003570	acyltransferase, putative	2.3.1.15	glycerophospholipid metabolism	**2**	<2	<2	3.08E-13
TGME49_010260	glycerol-3-phosphate dehydrogenase, putative	1.1.1.8	glycerophospholipid metabolism	**188**	14	32	6.84E-13
TGME49_082230	sulfate adenylyltransferas-adenylylsulfate kinase, putative	2.7.7.4	purine metabolism	**52**	5	10	7.38E-13
TGME49_107030	purine nucleoside phosphorylase	2.4.2.3	pyrimidine salvage pathway	**12**	2	3	1.11E-16
TGME49_003720	vitamin K epoxide reductase complex subunit 1, putative	1.1.4.1	terpenoid-quinone biosynthesis	9	21	**23**	1.63E-08
TGME49_002140	methylglutaconyl-CoA hydratase, putative	4.2.1.18	valine, leucine and isoleucine degradation	**5**	2	2	1.02E-06
TGME49_005430	isovaleryl-CoA dehydrogenase, putative	1.3.99.10	valine, leucine and isoleucine degradation	8	**13**	6	2.49E-10
TGME49_015250	thiamin pyrophosphokinase, putative	2.7.6.2	vitamin biosynthesis	<2	<2	<2	1.03E-09
***cell rescue, defence and virulence***							
TGME49_005660	F5/8 type C domain-containing protein (CCp1A)	-	-	**114**	11	14	1.00E-30
TGME49_023480	thrombospondin type 1 domain-containing protein	-	-	**24**	<2	<2	6.35E-07
TGME49_056040	LCCL domain-containing protein (CCp5B)	-	-	**343**	24	32	1.11E-15
TGME49_058370	Rhoptry kinase family protein ROP28	-	-	<2	**46**	23	2.51E-12
TGME49_063410	scavenger receptor protein TgSR2	-	-	**144**	12	12	2.22E-15
TGME49_064070	antigenic protein, putative (CCp1B)	-	-	**76**	7	8	1.10E-10
TGME49_067410	scavenger receptor protein TgSR1	-	-	**306**	26	29	2.21E-08
TGME49_027150	glutaredoxin, putative	-	detoxification of oxygen radicals	-3	-3	-3	6.84E-05
TGME49_116190	superoxide dismutase, putative	1.15.1.1	detoxification of oxygen radicals	<2	**589**	470	3.76E-12
TGME49_009920	PAN domain-containing protein	-	host cell attachment/invasion	<2	**557**	142	2.33E-14
TGME49_032400	PAN domain-containing protein	-	host cell attachment/invasion	<2	**44**	8	7.77E-15
TGME49_038460	SRS22B	-	host cell attachment/invasion	<2	<2	<2	8.95E-05
TGME49_058550	SRS28 (SporoSAG)	-	host cell attachment/invasion	2	**323**	312	1.44E-14
TGME49_058810	SRS27B	-	host cell attachment/invasion	<2	**153**	46	4.84E-09
TGME49_065120	hypothetical protein, conserved (RON2 iso2)	-	moving junction formation	<2	**28**	13	8.17E-08
TGME49_094400	hypothetical protein (RON2 iso 1)	-	moving junction formation	n.a.	n.a.	n.a.	5.89E-06
TGME49_115730	apical membrane antigen, putative (AMA1 iso1)	-	moving junction formation	<2	**16**	4	4.98E-12
***cell cycle and DNA processing***							
TGME49_006400	DNA photolyase, putative	4.1.99.3	DNA repair	34	**252**	99	1.82E-10
***protein fate***							
TGME49_016150	oligoendopeptidase F, putative	-	-	**213**	25	52	1.21E-11
TGME49_031060	subtilase family serine protease, putative	3.4.21.66	-	<2	**2**	<2	3.95E-07
TGME49_034950	protein kinase (incomplete catalytic triad)	-	-	<2	2	**5**	2.22E-15
TGME49_044620	NEK kinase	2.7.12.2	-	**2**	<2	<2	1.11E-10
TGME49_046800	acylamino-acid-releasing enzyme, putative	3.4.19.1	-	<2	-2	<2	2.10E-10
TGME49_047240	ubiquitin carboxyl-terminal hydrolase isozyme L5, putative	3.4.19.12	deubiquitination pathway	**3**	-2	<2	7.70E-11
TGME49_066780	protein kinase (incomplete catalytic triad)	-	-	**3**	<2	<2	4.22E-14
TGME49_089240	DnaJ domain-containing protein	1.3.1.38	-	<2	<2	<2	4.52E-11
TGME49_095640	endothelin-converting enzyme, putative	3.4.24.71	-	**27**	3	4	3.79E-12
TGME49_107640	CMGC kinase, CK2 family	2.7.12.2	-	**140**	19	27	2.15E-12
TGVEG_058750	protein kinase domain-containing protein, putative	2.7.11.11	-	n.a.	n.a.	n.a.	8.47E-07
***cellular transport, transport facilities and transport routes***							
TGME49_065230	electron transfer flavoprotein-ubiquinone oxidoreductase, putative	1.5.5.1	-	**8**	3	3	1.13E-12
TGME49_114330	ABC transporter, putative	3.6.3.44	-	**31**	13	12	9.50E-05
***protein synthesis***							
TGME49_020300	ribosomal protein L15, putative	-	translation	<2	<2	<2	9.08E-05
***biogenesis of cellular components***							
TGME49_043720	peroxisomal biogenesis factor 11 domain-containing protein	-	peroxisome fission	15	**22**	20	1.25E-05
TGME49_086250	oocyst wall protein, putative (OWP6)	-	oocyst environmental resistance	**44**	7	12	6.83E-10
***other***							
TGGT1_064840	hypothetical protein	-	-	n.a.	n.a.	n.a.	7.85E-08
TGGT1_071710	hypothetical protein, conserved	-	-	n.a.	n.a.	n.a.	5.11E-12
TGGT1_090830	hypothetical protein, conserved	-	-	n.a.	n.a.	n.a.	2.89E-05
TGGT1_099390	hypothetical protein, conserved	-	-	n.a.	n.a.	n.a.	7.99E-14
TGGT1_122010	hypothetical protein, conserved	-	-	n.a.	n.a.	n.a.	6.77E-14
TGGT1_122320	hypothetical protein, conserved	-	-	n.a.	n.a.	n.a.	4.09E-13
TGME49_002100	hypothetical protein	-	-	**847**	425	583	1.36E-07
TGME49_002110	hypothetical protein	-	-	**1163**	321	751	7.05E-11
TGME49_003230	hypothetical protein	-	-	11	**42**	27	3.05E-09
TGME49_003890	hypothetical protein	-	-	29	**150**	77	1.80E-09
TGME49_007250	hypothetical protein	-	-	**155**	15	28	7.55E-07
TGME49_009470	hypothetical protein	-	-	**476**	63	155	7.17E-10
TGME49_009810	hypothetical protein	-	-	<2	**30**	11	2.86E-12
TGME49_012030	hypothetical protein	-	-	<2	**9**	5	8.40E-11
TGME49_016970	hypothetical protein, conserved	-	-	-5	**2**	<2	1.44E-14
TGME49_020280	SCP-like domain-containing protein	-	-	n.a.	n.a.	n.a.	1.00E-30
TGME49_023430	hypothetical protein	-	-	<2	**66**	6	1.23E-09
TGME49_023830	fasciclin domain-containing protein	-	-	n.a.	n.a.	n.a.	1.50E-08
TGME49_023900	hypothetical protein	-	-	**4**	<2	<2	4.63E-05
TGME49_025120	rRNA processing protein, putative	-	-	n.a.	n.a.	n.a.	1.00E-09
TGME49_026230	hypothetical protein	-	-	<2	**21**	**21**	3.78E-08
TGME49_028240	hypothetical protein	-	-	<2	<2	<2	2.11E-13
TGME49_030530	hypothetical protein	-	-	**220**	22	33	1.99E-10
TGME49_030540	hypothetical protein	-	-	**53**	6	10	4.33E-08
TGME49_031390	hypothetical protein	-	-	**618**	69	138	1.46E-09
TGME49_037080	hypothetical protein	-	-	**1315**	582	905	1.56E-10
TGME49_038160	hypothetical protein	-	-	<2	**252**	78	1.03E-13
TGME49_038910	hypothetical protein	-	-	n.a.	n.a.	n.a.	3.50E-05
TGME49_040610	hypothetical protein, conserved	-	-	**65**	5	3	3.57E-08
TGME49_044410	hypothetical protein	-	-	n.a.	n.a.	n.a.	1.67E-07
TGME49_048170	hypothetical protein	-	-	<2	**4**	2	3.33E-14
TGME49_048230	hypothetical protein	-	-	<2	<2	<2	7.93E-05
TGME49_054760	hypothetical protein	-	-	**153**	126	80	1.12E-07
TGME49_055380	hypothetical protein	-	-	**917**	150	228	8.39E-12
TGME49_057690	hypothetical protein	-	-	**16**	3	3	2.10E-10
TGME49_058200	glutamic acid-rich protein, putative	-	-	<2	**48**	31	6.43E-09
TGME49_058380	hypothetical protein	-	-	<2	<2	<2	6.17E-05
TGME49_059670	von Willebrand factor type A domain-containing protein	-	-	<2	**257**	41	2.22E-15
TGME49_062470	beta antigen, putative	-	-	**658**	129	203	5.09E-05
TGME49_065130	hypothetical protein	-	-	<2	<2	<2	3.30E-11
TGME49_067980	hypothetical protein	-	-	<2	**9**	5	2.01E-07
TGME49_068880	hypothetical protein	-	-	-19	**-3**	-4	3.51E-09
TGME49_069350	hypothetical protein	-	-	<2	<2	<2	3.29E-11
TGME49_070950	hypothetical protein	-	-	15	238	**348**	5.74E-08
TGME49_071490	hypothetical protein	-	-	198	148	**282**	6.91E-13
TGME49_073510	hypothetical protein	-	-	<2	**79**	41	6.66E-15
TGME49_073670	hypothetical protein	-	-	-2	<2	<2	3.79E-05
TGME49_073700	hypothetical protein	-	-	**260**	27	52	3.42E-08
TGME49_076860	late embryogenesis abundant domain-containing protein	-	-	<2	**556**	390	2.22E-15
TGME49_076870	LEA1 protein, putative	-	-	8	**497**	146	2.03E-12
TGME49_078120	SCP-like extracellular domain-containing protein	-	-	<2	**96**	66	5.81E-09
TGME49_078340	hypothetical protein	-	-	2	**4**	<2	6.67E-07
TGME49_079440	hypothetical protein, conserved	-	-	**161**	14	26	4.43E-09
TGME49_080430	hypothetical protein	-	-	**1057**	220	376	1.47E-07
TGME49_085690	notch (DSL) domain-containing protein	-	-	**121**	15	21	8.51E-12
TGME49_087840	hypothetical protein	-	-	<2	<2	<2	3.00E-14
TGME49_088220	hypothetical protein	-	-	**52**	51	13	1.00E-30
TGME49_089740	hypothetical protein	-	-	<2	-4	<2	3.44E-07
TGME49_090610	hypothetical protein	-	-	**557**	38	42	5.26E-08
TGME49_091840	hypothetical protein	-	-	**260**	15	26	1.00E-30
TGME49_093780	hypothetical protein	-	-	-60	<2	<2	2.56E-06
TGME49_094600	hypothetical protein	-	-	15	506	**557**	3.19E-13
TGME49_097280	hypothetical protein, conserved	-	-	<2	**453**	139	2.63E-13
TGME49_097410	hypothetical protein	-	-	**187**	31	72	1.38E-08
TGME49_106050	hypothetical protein	-	-	**89**	14	30	1.11E-15
TGME49_106500	hypothetical protein	-	-	<2	<2	<2	6.28E-11
TGME49_109530	hypothetical protein	-	-	<2	**3**	<2	1.96E-11
TGME49_109540	hypothetical protein	-	-	<2	<2	<2	2.57E-10
TGME49_110160	piwi-PAZ domain-containing protein (TgAgo)	-	gene silencing	**21**	7	5	7.45E-12
TGME49_116890	hypothetical protein	-	-	**46**	5	9	5.18E-12
TGME49_119890	hypothetical protein	-	-	-14	**53**	33	1.08E-09
TGME49_120280	hypothetical protein	-	-	3	**16**	12	7.52E-12
TGME49_120290	hypothetical protein	-	-	**8**	<2	<2	4.68E-11
TGME49_120500	patched transmembrane domain-containing protein	-	-	**12**	<2	<2	1.37E-07
TGME49_120710	hypothetical protein	-	-	<2	**9**	4	2.15E-07
TGVEG_029770	hypothetical protein	-	-	n.a.	n.a.	n.a.	6.87E-06
TGVEG_051660	hypothetical protein, conserved	-	-	n.a.	n.a.	n.a.	8.83E-13
TGVEG_058740	hypothetical protein, conserved	-	-	n.a.	n.a.	n.a.	2.22E-16
TGVEG_060360	hypothetical protein	-	-	n.a.	n.a.	n.a.	1.28E-11
TGVEG_095590	hypothetical protein	-	-	n.a.	n.a.	n.a.	3.27E-11

^a^Fold change values refer to differences between the mRNA levels of unsporulated (day 0) or sporulated (day 4 and day 10) oocysts compared to day 2 in vitro tachyzoites of strain M4 [34].

Negative values indicate a downregulation in oocysts with respect to tachyzoites. Peak fold change values ≥2 are bolded. n.a., not available.

### Functional role of molecules belonging to the POSP subset

Compared to the whole oocyst/sporozoite dataset, the POSP class exhibited a significant overrepresentation of hypothetical proteins (45% versus 35%), probably reflecting a defect in the annotation of oocyst/sporozoite proteins caused by the paucity of molecular studies on this parasite stage. Seventy-two out of 154 POSP (47%) were assigned a MIPS functional category, with proteins involved in metabolism (28) constituting the largest class, followed by molecules implicated in cell rescue, defence and virulence (17), energy production (12) and protein fate (11).

### Central carbon metabolism

Our proteomic analysis showed that *T. gondii* oocysts express the entire enzymatic complements involved in glycolysis/gluconeogenesis and the tricarboxylic acid (TCA) cycle, the two main components of the central carbon metabolism of living organisms. Interestingly, VEG oocysts differed from tachyzoites for the abundant expression of two enolase isoforms, ENO1 and ENO2 [36]. So far, these two glycolytic enzymes were shown to be expressed in a mutually exclusive, stage-specific fashion in bradyzoites and tachyzoites, respectively, with only minimal amounts of ENO1 detected in tachyzoites by two proteomic studies [13,20]. While abundantly represented in our dataset, ENO1 was not identified in fully sporulated M4 oocysts [25], strongly suggesting that the expression of this bradyzoite-type enolase is developmentally regulated during oocyst maturation and is most likely specific to the unsporulated form. Also the TCA cycle comprises reactions catalyzed by two enzyme isoforms, e.g., isocitrate dehydrogenase (TGME49_066760, TGME49_113140) and succinate dehydrogenase (TGME49_015280, TGME-49_015590), which were all detected by proteomics in both tachyzoites and VEG oocysts. In addition, our analysis identified two differentially expressed isoforms of citrate synthase, TGME49_068890, detected also in tachyzoites and TGME49_003110, which is specific to the oocyst stage. Citrate synthase is a rate-determining enzyme converting oxaloacetate into citrate in the first step of the TCA cycle, therefore, the stage-specific expression of a second isoform may have important regulatory effects during oocyst development. Likewise, we found that the oocyst possesses two 50% identical isoforms of phosphoenolpyruvate carboxykinase, which provides a crucial link between the TCA cycle and gluconeogenesis by transforming oxaloacetate into phosphoenolpyruvate. One of the two isoforms, TGME49_089930, was uniquely identified in the oocyst and is encoded by a gene showing 30-fold transcriptional increase in unsporulated oocysts compared to tachyzoites. This enzyme might fuel the gluconeogenetic pathway and the eventual production of amylopectin [37], which represents an important energy source for sporulating oocysts and sporozoites.

### Amino acid metabolism

The POSP subset included key enzymes for the synthesis of 6 non essential amino acids of *T. gondii*, i.e., proline (TGME49_069110), alanine (TGME49_115260, TGME-49_003500), threonine (TGME49_016640), cysteine (TG-ME49_059180, TGME49_078910, TGME49_112930), lysine (TGME49_005420) and tyrosine (TGME49_08-7510, TGME49_012740). The two forms of alanine dehydrogenase (EC 1.4.1.1), which catalyses the unidirectional conversion of pyruvate into l-alanine, share 39% sequence identity and differ for their predicted subcellular localization, cytoplasmic for TGME49_115260 and mitochondrial for TGME49_003500. We detected also two 98% identical isoforms of tyrosine hydroxylase (TGME49_087510, TGME49_012740) possessing a bifunctional activity able to catabolize phenylalanine to tyrosine and tyrosine to 3,4-dihydroxy- l-phenylalanine (l-DOPA) [38]. The presence of these two enzymes in the oocyst stage may be important not only for de novo tyrosine synthesis, but also for the supply of l-DOPA, which was shown to be a component of the oocyst wall of *Eimeria maxima*[39], an avian coccidian parasite related to *T. gondii*. The oocyst differs from the tachyzoite also for the expression of three distinct enzymes involved in the biosynthesis of cysteine. This important amino acid can be produced either from l-serine, through the successive action of cystathionine β-synthase (TGME49_059180) and cystathionine β-lyase (TGME49_112930), or from O-acetylserine, through the fixation of inorganic sulphide by a plant/bacterial-like O-acetylserine (thiol) lyase (TGME49_078910). In contrast with previous views, current genomic data indicate that *T. gondii* is not a threonine auxotroph but has the potential to synthesize this amino acid using homoserine as precursor. Our data showed that a predicted enzyme linked to this pathway, homoserine kinase (TGME49_016640), is indeed expressed in the oocyst stage.

### Branched amino acid degradation

The analysis of the POSP subset revealed that also the degradation pathway of the branched amino acids valine, leucine and isoleucine, which represents an important source of acetyl-CoA, is more extensively covered in the oocyst than in the tachyzoite. Two POSPs mapping to this mitochondrial pathway correspond to enzymes specifically involved in leucine degradation, i.e., isovaleryl-CoA dehydrogenase (TGME49_005430; EC 1.3.99.10) and methyl-glutaconyl-CoA hydratase (TGME49_002140; EC 4.2.1.18), whereas a third enzyme, 2-methylbutyryl-CoA dehydrogenase (TGME49_066270), is common to the pathways leading to valine and isoleucine degradation and fatty acid β-oxidation.

### Fatty acid metabolism

The POSPs include several enzymes implicated in fatty acid degradation, also known as β-oxidation. This important energy-producing process consists of successive rounds of four reactions each, during which one acetyl-CoA two-carbon unit is released at each cycle from even-chain fatty acids. The acetyl-CoA generated enters the TCA cycle, where it is further oxidized to fuel ATP synthesis via oxidative phosphorylation. The genome of *T. gondii* encodes all enzymes required for this process and six of them are represented in the POSP subset, i.e., two isoforms of acyl-CoA dehydrogenases (TGME49_047500, TGME49_115480; EC 1.3.3.6), 2-methylbutyryl-CoA dehydrogenase (TGME-49_066270; EC 1.3.8.1), 2,3-enoyl-CoA hydratase (TGME49_042390; EC 4.2.1.17), 3-hydroxyacyl;-CoA dehydrogenase (TGME49_034570; EC 1.1.1.35) and 2,4-dienoyl-CoA reductase (TGME49_026300; EC 1.3.1.34). Interestingly, four of these enzymes share the presence of a peroxisomal targeting signal, including TGME49_047500, TGME-49_042390 and TGME49_034570, which catalyse the first three reactions of the β-oxidation cycle, respectively, and TGME49_026300, which plays an auxiliary role in the degradation of unsaturated fatty acids in animal peroxisomes. Beside the peroxisomal TGME49_047500 mentioned above, two additional POSPs belong to the class of FAD-dependent acyl-CoA dehydrogenases involved in the first step of β-oxidation, i.e., TGME49_115480 and TGME-49_066270. The two enzymes lack obvious organelle targeting signals and show substrate preference for medium- and short-chain fatty acids, respectively. Overall, these results indicate that, unlike tachyzoites, *T. gondii* oocysts rely on fatty acid breakdown to fuel the TCA cycle through the production of acetyl-CoA. Furthermore, the profile of β-oxidation proteins identified strongly suggests that, like in mammals, also in *T. gondii* this process takes place in both the peroxisomes and the mitochondria, with the two compartments being presumably involved in the degradation of very long- and medium/short-chain fatty acids, respectively.

### Amylopectin metabolism

Like other apicomplexan parasites forming environmentally resistant oocysts, e.g., *Eimeria* spp. and *Cryptosporidium* spp., *T. gondii* stores glucose in cytoplasmic granules of amylopectin, a polysaccharide consisting of long linear chains of glucose units linked by α-1,4-glycosidic bonds, with occasiona1α-1,6-glycosidic bonds providing a branching point [37]. While virtually absent in tachyzoites, amylopectin is abundant in unsporulated oocysts, sporozoites and bradyzoites [40], which use it as energy source. Amylopectin degradation in *T. gondii* depends on the phosphorylase TGME49_110670 (EC 2.4.1.1), identified by proteomics in both oocysts and tachyzoites, which liberates monomers of glucose-1-phosphate by cleaving α-1,4-glycosidic bonds up to four glucose residues from a branching point. At this stage, further glucose mobilization requires a debranching step, provided by glucanotransferase (EC 2.4.1.25) and glucosidase (EC 3.2.1.33) activities. The former transfers three glucose residues from a four-residue amylopectin branch to a nearby α-1,4 chain, whereas the second cleaves the remaining α-1,6 linkage, producing a glucose unit. These two activities are provided by bifunctional debranching enzymes in mammals and yeast and by two distinct enzymes in most bacteria. We found that *T. gondii* oocysts share with tachyzoites the bifunctional debranching enzyme TGME49_026910, but uniquely express a bacterial-like 4-α-glucanotransferase (TGME-49_071210), whose cognate microarray mRNA levels are 254-fold higher in day 4 oocysts than in tachyzoites. The stage-specific expression of this enzyme has the potential to increase the rate of amylopectin debranching and glucose mobilization, supporting the extraordinary oocyst’s energy demand associated with the sporulation process.

### Purine metabolism

Two enzymes linked to purine metabolism were identified exclusively in the oocyst. The first is purine nucleoside phosphorylase (PNP) (TGME49_107030), which converts exogenously acquired inosine and guanosine into hypoxanthine and guanine, respectively, thus providing a source of nucleobases for the synthesis of purine nucleotides. In accordance with the strict dependence of *T. gondii* on external supplies of purine nucleobases and nucleosides, the expression of PNP is likely an adaptative response maximising the incorporation of purines salvaged from the host cell before oocyst shedding. The second enzyme related to purine metabolism is the bifunctional sulfate adenylyltransferase-adenylylsulfate kinase (TGME49_082230), which catalyses the fixation of inorganic sulfate into the sulfuryl group donor molecule 3’-phosphoadenylyl-sulfate.

### Glycerophospholipid and vitamin metabolism

Two members of the POSP subset are related to glycerophospholipid metabolism. The first protein (TGME-49_010260) is a glycerol-3-phosphate dehydrogenase, one of the key enzymes of the glycerol phosphate shuttle system for the electron transport across the mitochondrial membrane. Interestingly, this molecule appears to be a stage-specific variant of TGME49_107570, a closely related form of glycerol-3-phosphate dehydrogenase expressed in tachyzoites and not identified in the present study. Likewise, TGME49_003570 is a putative glycerol-3-phosphate acyltransferase distantly related to a second form of the enzyme so far identified only in tachyzoites. Concerning vitamin biosynthesis, two enzymes lacking proteomic evidence in tachyzoites were identified, i.e., thiamin pyrophos-phokinase (TGME49_015250), which converts host-derived thiamine into its metabolically active form vitamin B1, and the subunit 1 (TGME49_003720) of the enzyme complex vitamin K epoxide reductase, which is responsible for the production of the co-factor vitamin K hydroquinone.

### Cell rescue, defence and virulence

**Figure 5:**
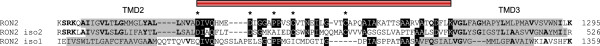
**Amino acid sequence variability in the AMA1-binding site of RON2 paralogs. **The amino acid sequence of the AMA1-binding site of TgRON2 (red bar) was aligned with the corresponding regions of TgRON2 iso1 (TGME49_094400) and TgRON2 iso2 (TGME49_065120). The C-terminal transmembrane domains (TMD2 and TMD3) of the three paralogs are highlighted in grey. Conserved amino acids within the AMA1-binding sequence are framed in black, those lying in the flanking regions are bolded. Asterisks indicate RON2 amino acid residues playing a crucial role in AMA1 binding [46].

This relatively heterogeneous functional category is well represented in the POSP subset and includes proteins implicated in host cell attachment/invasion (10), components of the parasite antioxidant system (2) and members of the LCCL family (5). Proteins of the attachment/invasion subgroup include the rhoptry kinase family [41] protein ROP28 and 3 members of the SRS superfamily [27], i.e., SRS22B, SRS27B and the major sporozoite surface antigen SRS28 (SporoSAG) [9]. Additionally, our analysis identified a thrombospondin-related (TGME49_023480) and 2 PAN domain-containing (TGME49_009920, TGME49_032400) proteins showing domain architectures resembling those of known micronemal adhesins of *T. gondii*. Interestingly, invasion-related POSPs include also paralogous versions of the mutually interacting MJ proteins AMA1 [42] and RON2 [43,44], denominated AMA1 iso1 (TGME49_115730), RON2 iso1 (TGME49_094400) and RON2 iso2 (TGME49_065120) [45]. Recently, a crystallographic analysis mapped the AMA1-binding site of RON2 to a C-terminal sequence comprised between two closely spaced transmembrane domains [46]. The alignment of this amino acid sequence with the equivalent regions of the RON2 paralogs (Figure 5) revealed a higher similarity to RON2 iso2 and the conservation in this protein of 4 amino acid residues crucial for AMA1-RON2 interaction. The co-expression of 2 AMA1 and 3 RON2 isoforms represents a very distinctive trait of sporozoites which, compared to tachyzoites, seem to have the potential to assemble different MJ types.

Previous studies [47,48] revealed the presence in *T. gondii* of an elaborated antioxidant network based on cytosolic, mitochondrial and apicoplast enzymes implicated in the detoxification of oxygen radicals. Our analysis identified 2 members of this network lacking tachyzoite proteomic evidence, i.e., the mitochondrial superoxide dismutase SOD3 (TGME49_116190) and a putative glutaredoxin (TGME49_027150). While the mRNA levels of the SOD3 gene are 470-fold higher in sporulated oocysts compared to thachyzoites, the gene encoding glutaredoxin TGME49_027150 appears to be equally expressed in all stages.

The third class of molecules lacking proteomic evidence in the tachyzoite consists of five proteins (TGME-49_005660, TGME49_064070, TGME49_056040, TGME-49_067410 and TGME49_063410) belonging to the so-called LCCL family, which is exclusive, among protozoan parasites, of the apicomplexa. In *T. gondii* this group of multi-modular proteins is composed by 7 members containing one or more copies of the LCCL domain (F. Spano, unpublished). Although the function of this protein class in *T. gondii* remains unknown, there is evidence that the plasmodial orthologs have a sexual stage-specific expression and are involved in oocyst/sporozoite maturation [49].

### Proteins belonging to other MIPS functional categories

Other functionally heterogeneous members of the POSP subset appear particularly relevant to oocyst biology. One example is the DNA photolyase TGME49_006400, an evolutionarily conserved enzyme that repairs DNA damage, i.e., pyrimidine dimers, caused by the exposure to ultraviolet light. The stage-specificity of this enzyme is consistent with the oocyst need to resist various environmental stresses, including ultraviolet radiation. Accordingly, the DNA photholyase transcript is 30–100 fold more abundant throughout oocyst maturation compared to tachyzoites and bradyzoites. Another protein strictly related to oocyst resistance is the oocyst wall protein 6 (TgOWP6; TGME49_086250). As we showed previously [10], TgOWP6 belongs to a family of seven structurally related cysteine-rich proteins named TgOWP1-7. Using specific antibodies, TgOWP1-3 were shown to be abundantly expressed in the oocyst wall of *T. gondii*[10], yet in the present study TgOWP6 was the only member of this protein class to be identified, probably reflecting difficulties in solubilizing TgOWPs’ monomers engaged in multimeric complexes.

## Discussion

We reported herein on the identification by mass spectrometry of 1615 proteins expressed in the oocyst of *T. gondii,* a developmental stage which has been scarcely characterized at the molecular level, despite its fundamental role in disease transmission. The present work adds to a very recently published proteomic analysis of *T. gondii* oocysts of strain M4 [25]. Both studies employed 1-D gel electrophoresis LC MS-MS, yet they differed for the parasite strain analysed, i.e., VEG (genotype III) versus M4 (genotype II), for the modality of protein extraction and fractionation, and principally for the maturation stage of the oocyst samples, which consisted of partially or fully sporulated oocysts, respectively. While the presence in our sample of both unsporulated and sporulated oocysts hampered the association of protein expression with a well defined oocyst maturation stage, it enabled the proteomic sampling of a wider range of proteins, including those expressed exclusively, or more abundantly, prior to sporulation. Excluding the proteins identified with a single peptide in just one experiment, the VEG oocyst dataset exceeded that of strain M4 by 510 proteins, of which 73 lacked proteomic evidence in the tachyzoite and were identified here for the first time.

Although the coverage of the oocyst proteome provided by our analysis could not be determined, the considerable size of the produced dataset offered a representative view of the protein complement expressed during oocyst development and sporozoite differentiation. One of the major implications of the present study was to shed light for the first time on the metabolic organization of the oocyst/sporozoite, unveiling stage-specific differences with respect to the extensively studied tachyzoite. Numerous metabolic routes were identified underlying the metabolism of a wide range of molecules, including lipids, carbohydrates, amino acids, nucleotides and vitamins. Consistent with the resolution limit inherent to a single-platform, global proteomic analysis and considering that protein expression levels may range across many orders of magnitude, several metabolic pathways were not entirely covered by our study. This implies that establishing the non functionality of a given metabolic route in the oocyst will require a higher number of identifications. Nevertheless, the absence from both VEG and M4 [25] datasets of the entire enzymatic complements specific of isoprenoids (8 enzymes) and shikimate (5 enzymes) biosynthesis suggests that in the oocyst these pathways may be markedly downregulated or missing.

We found that *T. gondii* oocysts possess the full complement of both glycolytic and TCA cycle enzymes, differing from tachyzoites for the expression of alternative isoenzymes of enolase (ENO1), citrate synthase and phosphoenolpyruvate carboxykinase, which connects the two pathways by fueling gluconeogenesis. The relative contribution of glycolysis and TCA cycle to energy production in *T. gondii* is still an open issue. Previous works compared the in vitro activity of select enzymes involved in either of the two pathways, highlighting stage-specific differences between tachyzoites and bradyzoites. Biochemical data revealed that in bradyzoites glycolysis largely predominates over the TCA cycle, suggesting that this latent parasite stage lacks a functional electron transport chain [50]. Conversely, tachyzoites rely on both pathways, with glycolysis playing a major role in extracellular parasites by supplying most of the energy required for gliding motility and invasion [51,52], whereas oxidative phosphorylation is important for ATP homeostasis in intracellular tachyzoites [53]. Importantly, there is evidence that in *T. gondii* and *Plasmodium* spp. the TCA cycle is not coupled to glycolysis, as the pyruvate dehydrogenase complex, which catalyses pyruvate conversion into acetyl-CoA, is not typically localized to the mitochondrion, but is specific to the apicoplast [54,55]. This striking feature raises the question as to how *T. gondii* generates the mitochondrial acetyl-CoA to be fed into the TCA cycle. Our proteomic data indicated a very plausible explanation, pointing to a major involvement of two acetyl-CoA-producing pathways alternative to glycolysis, i.e., the β-oxidation of fatty acids and the degradation of branched amino acids. In fact, we identified all enzymes of the fatty acid β-oxidation route and the majority of those involved in the catabolism of valine, leucine and isoleucine, with several enzymes of both pathways lacking proteomic evidence in tachyzoites. The stage-specific usage of these two mitochondrial pathways is consistent with the peculiar biology of the oocyst, which must undergo the energy-demanding process of sporulation and survive in the environment for prolonged periods, without a continuous supply of host-derived nutrients. As an alternative to fatty acid and branched amino acid degradation, intracellular tachyzoites presumably fuel the TCA cycle using the enzyme acetyl-CoA synthetase (TGME49_066640), which could produce acetyl-CoA from the acetate scavenged from host cell mitochondria recruited to the parasitophorous vacuole. Comparative genomic analysis showed that apicomplexan parasites exhibit markedly different patterns of amino acid auxotrophy, with *T. gondii* encoding a complement of enzymes for the synthesis of 12 out of 20 amino acids, as opposed to 6 in *Plasmodium*, 4 in *Cryptosporidium* and 3 in *Theileria*[56]. We provided proteomic evidence that the capacity of de novo amino acid biosynthesis is developmentally modulated in *T. gondii*, with oocysts showing a greater commitment than tachyzoites, a feature that is in accordance with the long-term permanence of the oocyst in the extracellular environment.

The vast majority of the protein identifications reported herein were corroborated by mRNA expression data, 94% of which were derived from microarray analysis of M4 oocysts collected at day 0, 4 or 10 of sporulation [34]. In the present study, we employed a heterogeneous preparation of oocysts at various maturation stages, therefore a complete comparison between VEG proteomic and M4 microarray results was not feasible. However, 62 out of 1304 (4.7%) VEG proteins were encoded by genes not exceeding the 25^th^ microarray percentile in M4 oocysts at all three sporulation time points. This evidence indicates that in our study a significant, and probably underestimated, proportion of proteins was successfully identified despite cognate mRNA levels close to baseline. Regarding the POSP subset, we found that of 134 proteins supported by microarray evidence in strain M4, approximately 30% were either associated with mRNA levels differing by less than 2-fold between oocysts and tachyzoites (9.7%) or encoded by transcripts whose relative abundance in tachyzoites was elevated, being comprised between the 50^th^ and the 100^th^ percentile (20.8%). These data are in evident contrast with the absence of these proteins from the extensively covered proteome expressed by the tachyzoite [32] and argue for the existence in *T. gondii* of post-transcriptional control mechanisms of protein expression, as already reported in other protozoan parasites [57-59]. In accordance with this hypothesis, we have previously shown that the genes TgOWP1, TgOWP2 and TgOWP3 are transcribed in both VEG oocysts and tachyzoites, yet the encoded oocyst wall proteins are not expressed in the latters [10]. Sequence analysis of RT-PCR products showed that tachyzoites differed from the oocyst for the presence of multiple mRNA forms of TgOWP1-3 in which one or more introns were retained, abolishing the coding potential of the immature transcripts. The same phenomenon was observed also for the SporoSAG gene (F. Spano, unpublished), pointing to the inhibition of pre-mRNA splicing as one of the strategies underlying stage-specific protein expression in *T. gondii*.

The molecular bases of host cell invasion by the sporozoite of *T. gondii* are virtually unknown, mostly due to the difficulty of preparation and the transitory nature of this infectious stage, which rapidly differentiates into a tachyzoite inside the host cell. It can be reasonably assumed that sporozoites and tachyzoites utilize the same invasion machinery, yet there is evidence that the formers express stage-specific invasion-related proteins [9,25], exemplified here by SRS22B, SRS27B, SRS28, ROP28 and by isoforms of AMA1 and RON2. In addition, two of the POSPs identified, TGME49_032400 and TGME49_009920 [60], are valid MIC candidates, as they both possess a leader peptide followed by tandem copies of the PAN domain, previously described in the microneme proteins TgMIC4 of *T. gondii*[61] and EtMIC5 of *Eimeria tenella*[62]. Finally, it cannot be excluded that the POSP subset contains other invasion-related proteins, whose identification may be hampered by the low degree of phylogenetic conservation typical of ROPs and GRAs.

## Conclusions

We have described the results of a global proteomic analysis of the oocyst/sporozoite of *Toxoplasma gondii*, expanding significantly the coverage of the protein repertoire expressed by this important parasite stage. Using partially sporulated oocysts of the genotype III strain VEG, we generated a dataset of 1304 non reduntant proteins, accounting for ~18% of the total predicted proteome. Approximately 38% of the identifications corresponded to hypothetical proteins, whereas ~59% were classified according to the MIPS functional catalogue database. Importantly, we identified 154 proteins lacking preoteomic evidence in the extensively studied tachyzoite stage, which are likely to represent oocyst/sporozoite-specific proteins (POSPs). Their functional profile reflects the adaptation of the oocyst of *T. gondii* to the nutrient-poor and stressing extracellular environment, as shown by the preponderance of enzymes involved in metabolism (28) and energy production (12) or by the presence of a photolyase DNA repair enzyme. Compared to tachyzoites, oocysts were found to have a greater capability of de novo amino acid biosynthesis and to rely on the degradation of fatty acids and branched amino acids as sources of acetyl-CoA. In addition, our data indicated that *T. gondii* sporozoites and tachyzoites possess extensively overlapping repertoires of invasion-related proteins, yet sporozoites express stage-specific isoforms of AMA1 and RON2, two proteins essential for moving junction formation and host cell invasion. Finally, the comparison between proteomic and microarray data showed that the stage-specific expression of a significant fraction of the POSPs cannot be justified by different mRNA levels in oocysts and tachyzoites, suggesting the existence of post-transcriptional control mechanisms. The present work shed light for the first time on the metabolic organization of the oocyst of *T. gondii* and represents a further step toward the understanding of the fundamental biology of this important parasite stage. A major goal of future proteomic studies should be the elucidation of proteome dynamics during oocyst transition from the unsporulated to the metabolically active, sporulating state, until the development of infectious, metabolically quiescent, sporozoites.

## Methods

### Parasites

*Toxoplasma gondii* oocysts of the VEG strain (genotype III) were recovered from the feces of experimentally infected cats housed at the United States Department of Agriculture Laboratory, Beltsville, Maryland as described [63]. All cats used in experiments were handled using procedures approved by the Beltsville Area Animal Care and Use Committee, United States Department of Agriculture, USA. Sporulation was induced by incubating the oocysts in 2% sulfuric acid at room temperature for one week, successively keeping the parasites in the same solution at 4°C for prolonged storage. Oocyst were purified by sucrose floatation as described previously [1] and successively stored in 2% sulfuric acid. Tachyzoites of the VEG strain were cultured in human foreskin fibroblasts (HFF) in Dulbecco’s modified Eagle’s medium (DMEM) supplemented with 10% Nu-Serum (Gibco), 2 mM glutamine, 10 mM HEPES (N-2-hydroxyethylpiperazine N’-2-ethanesulfonic acid) and 20 μg/ml gentamicin.

### Protein extraction

Purified *T. gondii* oocysts were treated with 10% sodium hypochlorite for 20 min on ice, extensively washed with PBS and ruptured by shaking on a vortex with 400–600 μm glass beads (Sigma) for 5 min, to obtain free sporocysts. Sporozoite excystation was induced by incubating the broken oocysts with 1.5% sodium taurocholate at 37°C for 40 min. Following PBS washings, the parasite material was collected by centrifugation at 10000 g for 10 min and further processed for protein extraction. To produce total protein lysates (experiments 1 and 2), the parasite pellet was resuspended in urea/thiourea buffer (7 M urea, 2 M thiourea, 2% CHAPS, 1% ASB 1–14, 1% SB 1–12), freeze-thawed three times, supplemented with 200 mM DTT/1% SDS and shaked at 900 rpm for 3 h at 37°C. Following centrifugation at 16000 g for 30 min at 4°C, the supernatant was recovered and analysed by 1D gel electrophoresis LC MS/MS. To produce fractionated lysates (experiment 3), the parasite pellet was resuspended in 100 mM Tris–HCl pH 8.5, supplemented with protease inhibitors and 200 mM DTT. The sample was freeze–thawed three times, subjected to three cycles of 5 min in a water bath sonicator and centrifuged at 16000 g for 30 min at 4°C, prior to collection of the supernatant. The pellet was washed three times with 100 mM Tris–HCl pH 8.5, containing protease inhibitors, and the washings were pooled with the supernatant constituting the soluble fraction. Next, the pellet was resuspended in urea/thiourea buffer, freeze-thawed three times, supplemented with 100 mM DTT/2% SDS, subjected to three cycles of 5 min in a water bath sonicator and incubated for 2 h at 37°C with shaking at 900 rpm. Following centrifugation at 16000 g for 30 min at 4°C, the supernatant, representing the insoluble fraction, was collected and analysed by 1D gel electrophoresis LC MS/MS along with the soluble fraction. Tachyzoite proteins were extracted in urea/thiourea buffer as mentioned above, starting from VEG parasites released from infected HFF by passage through a 27-gauge syringe needle and subsequently filtered through 3-μm pore diameter filters.

### SDS-PAGE and in-gel digestion

From 50 to 75 μg of total protein extracts or soluble/insoluble protein fractions obtained from *T. gondii* oocysts were resolved by SDS-PAGE on 10% precast minigels (NuPAGE Novex Bis-Tris; Invitrogen), loading the samples in 1.2 cm-wide wells. Following staining with NuPage Colloidal Coomassie (Invitrogen), gel lanes were cut into 30 to 48 slices depending on protein band density. In-gel tryptic digestion was performed as previously described [64]. Briefly, gel slices were destained by washings in acetonitrile (ACN)/25 mM NH4CO3 (vol/vol), treated with 10 mM DTT (40 min at 56°C) and 55 mM iodoacetamide (30 min in the dark at RT) to reduce and alkylate cysteines, shrunken with ACN and rehydrated for 40 min on ice with a solution of 12,5 ng/μl trypsin (Promega) in 50 mM NH4CO3_._ Next, the trypsin solution was replaced with 25 mM NH4CO3 and protein digestion was carried out overnight at 37°C. Supernatants from individual gel slices were directly used for LC-MS/MS analysis.

### Liquid chromatography tandem mass spectrometry

Analysis by LC-MS/MS was performed using a nano-HPLC 3000 Ultimate (Dionex) connected in line to LTQ-XL linear ion trap (Thermo Fisher). Tryptic digests (20 μl) were firstly loaded on a C18 RP-precolumn (300 μm i.d.×5 mm; 5 μm particle size; 100 Å pore size; LC Packings-Dionex) and washed by the loading pump at 20 μl/min with buffer A (95% ACN, 0.1% FA) for 5 min and then by the micro pump at 0.3 μl/min on an homemade 10 cm × 75 μm-i.d. Silica PicoTip (8 ±1 μm) column (PicoTip Emitter, NewObjective) packed with Magic C18AQ (5 μm particle size; 200 Å pore size, Michrom Bioresources Inc.) for chromatographic separations. Peptides were eluted along a 50 min linear gradient from 4% to 50% of buffer B (95% ACN, 0.1 % FA) and electrosprayed directly into the mass spectrometer with a spray voltage of 1.65-1.75 kV and a capillary temperature of 180°C.

Data acquisition was performed in data-dependent mode to automatically switch between MS and MS2. Full-scan MS parameters settings was: automatic gain control value of 30000 ions; maximum injection time of 50 ms; m/z 400–2000 mass range. The five most intense ions were sequentially selected and fragmented in CID mode with the following parameters settings: isolation width of 2.0; automatic gain control value of 10000 ions; maximum injection time of 100 ms; m/z 50–2000 mass range; collision energy of 35%; minimum signal threshold of 200 counts; wide band activation on. A dynamic exclusion of ions previously sequenced within 30 sec was applied.

### Mass spectrometry data processing

Raw spectra were analysed by Bioworks Browser 3.3.1 using the Sequest 3.1 search engine against an indexed database containing all predicted tryptic peptides from *T. gondii* proteins (ToxoDB release 5.1) [65]. Carboamidomethylation of cysteines was specified as fixed modification and oxidation of methionine was set as variable modifications; mass tolerance was set to 1 Da for precursor ion and ±0.4 Da for fragment ions and a maximum of two missed cleavages was allowed. The minima criteria for protein identification were: peptide probability <1E-04; peptide *XCorr vs charge* = 1.5 for charge +1; 2 for charge +2; 2.5 for charge +3; 3 for charge +3; FDR <1%.

### Bioinformatics predictions

All proteins identified were assigned a subcellular localization combining the results of the general predictor WoLF PSORT (http://wolfpsort.org/) with those obtained with the softwares PlaMit (http://gecco.org.chemie.uni-frankfurt.de/plasmit/index.html), PATS (http://gecco.org.chemie.uni-frankfurt.de/pats/pats-index.php) and PTS1 (http://mendel.imp.ac.at/mendeljsp/sat/pts1/) for the identification of mitochondrial, apicoplast and peroxisomal targeting signals, respectively. Proteins were classified into functional categories according to the MIPS functional catalogue database (http://mips.helmholtz-muenchen.de/proj/funcatDB/search_main_frame.html) based on GO biological process annotations provided by ToxoDB, on KEGG Pathway database (http://www.genome.jp/kegg/) annotations and on BLASTP homology searches (http://blast.ncbi.nlm.nih.gov/).

### Production of recombinant proteins and western blot analysis

Recombinant fragments of the LCCL domain-containing proteins TGME49_056040 (amino acids 555–996) and TGME49_067410 (amino acids 353–682) and of the tyrosine-rich protein TGME49_119890 (amino acids 24–193) were produced in *Escherichia coli* (strain M15) as fusion products with an N-terminal tag of six histidines using the expression plasmid pQE30 (Qiagen). The following primers were used for PCR amplification of the target sequences from a *T. gondii* cDNA library of partially sporulated oocysts provided by Michael White (Montana State University): 056040.F (5^′^-GATCTGGATCCGGCAACGCCTACGCTGACTGCT-3^′^), 056040.R (5^′^-GTAGACTGCAGTTACGCAACCTCGTCAGAAATCTTT-3^′^); 067410.F (5^′^-AGTCAGGATCCGTAGCTATTGTGGATAGTCTGCCA-3^′^), 067410.R (5^′^-AGTCAAAGCTTTCATGCCATATCTCTCCTGGTGACTGG-3^′^); 119890.F (5^′^-ATCTGGATCCGAGACTGAGGAGGCGAAGTTG-3^′^), 119890.R (5^′^-AGATAAGCTTTTATCCACCGAAAAGATTGCCGAG-3^′^). The recombinant polypeptides were purified from total bacterial lysates by nickel affinity chromatography under denaturing conditions and successively dialysed against multiple changes of PBS, pH 7.2. To raise mouse antisera by intraperitoneal injection, BALB/c female mice were immunized three times (at 3–4 week intervals) with 50 μg of purified recombinant protein in complete (first immunization) or incomplete (first boost) Freund’s adjuvant. Individual sera were collected 2 weeks after the second boost (soluble antigen in PBS). The animal work has been authorized by the Italian Ministry of Health, according to the Legislative Decree 116/92 which implemented the European Directive 86/609/EEC on laboratory animal protection. Animals used in this study were housed at the Istituto Superiore di Sanità and treated according to Legislative Decree 116/92 guidelines.

Total protein extracts from VEG oocysts and tachyzoites were quantified with a Qubit fluorometer (Invitrogen) and equal amounts of the two samples were resolved by SDS–PAGE on 10% NuPage Novex Bis–Tris gels (Invitrogen). Following transfer to nitrocellulose, the blots were blocked with 5% w/v skimmed milk in 1X Tris Borate Saline Tween (TBST; 50 mM Tris pH 8.0, 150 mM NaCl, 1% Tween 20) and probed for 1 h with the appropriate mouse antiserum (1:1000) or with the anti-SAG1 mAb T41E5 (1:20000), kindly donated by J-F. Dubremetz, (University of Montpellier, France). After 30 min incubation with a goat anti-mouse IgG secondary antibody conjugated to horseradish peroxidase (BioRad), reactive protein bands were revealed by chemiluminescence with LiteAblot Plus (EuroClone, Italy).

## Abbreviations

ACN: Acetonitrile; DTT: Dithiothreitol; CoA: Coenzyme A; EST: Expressed Sequence Tag; GO: Gene Ontology; KEGG: Kyoto Encyclopedia of Genes and Genomes; LC-MS/MS: Liquid Chromatography Tandem Mass Spectrometry; l-DOPA: 3,4-Dihydroxy-L-Phenylalanine; MJ: Moving Junction; mRNA: messenger RNA; SDS: Sodium Dodecyl Sulfate; TgOWP: *Toxoplasma gondii* Oocyst Wall Protein; POSP: Putative Oocyst/Sporozoite-Specific Protein; RON: Rhoptry Neck Protein; SAGE: Serial Analysis of Gene Expression; TBST: Tris Borate Saline Tween; TCA: Tricarboxylic Acid.

## Competing interests

The authors declare that they have no competing interests.

## Authors’ contributions

FS conceived the study, analysed and interpreted the data and wrote the manuscript. E. Pizzi carried out data analysis and bioinformatic predictions. MP conceived the study, carried out data analysis and critically revised the manuscript. EP contributed to the experimental design and critically revised the manuscript. JPD carried out experimental infections of cats and oocyst purification. FF and LF performed mass spectrometry experiments and data analysis. AP performed oocyst purification, protein extraction and western blot analysis. All authors have read and approved the final version of the manuscript.

## Supplementary Material

Additional file 1: Figure S1 Electrophoretic profiles of fractionated oocyst/sporozoite proteins of *T. gondii*. In experiment 3, Tris-soluble and Tris-insoluble protein samples were resolved by SDS-PAGE on a 10% acrylamide gel under reducing conditions and processed for LC-MS/MS analysis. Protein bands were visualized by staining with colloidal Coomassie. The arrows indicate the positions of representative gel slices. Click here for file

Additional file 2: Table S1 Complete dataset of *T. gondii *oocyst/sporozoite proteins identified in this study. The table includes the list of the 1615 VEG oocyst proteins identified in the present study. Each protein is identified by the gene ID (column A) and annotation (column B) provided by ToxoDB 7.1. Columns C-E report Bioworks Browser 3.3.1 parameters, the number of peptides indicated in column E refers to the best identification. Column F reports the number of experiments in which each protein was identified. Column G indicates the predicted molecular weight of full length polypeptides. Asterisks indicate two proteins (TGGT1_064110 and TGGT1_064850) identified with a single peptide shared by other *T. gondii* proteins, whose IDs and annotations are reported at the bottom of the table. Click here for file
