# Supplementary figures and images for: Global proteomic analysis of the oocyst/sporozoite of Toxoplasma gondii reveals commitment to a host-independent lifestyle
Source: BMC Genomics. 2013 Mar 15;14:183. doi: 10.1186/1471-2164-14-183 (PMC3616887; doi:10.1186/1471-2164-14-183)

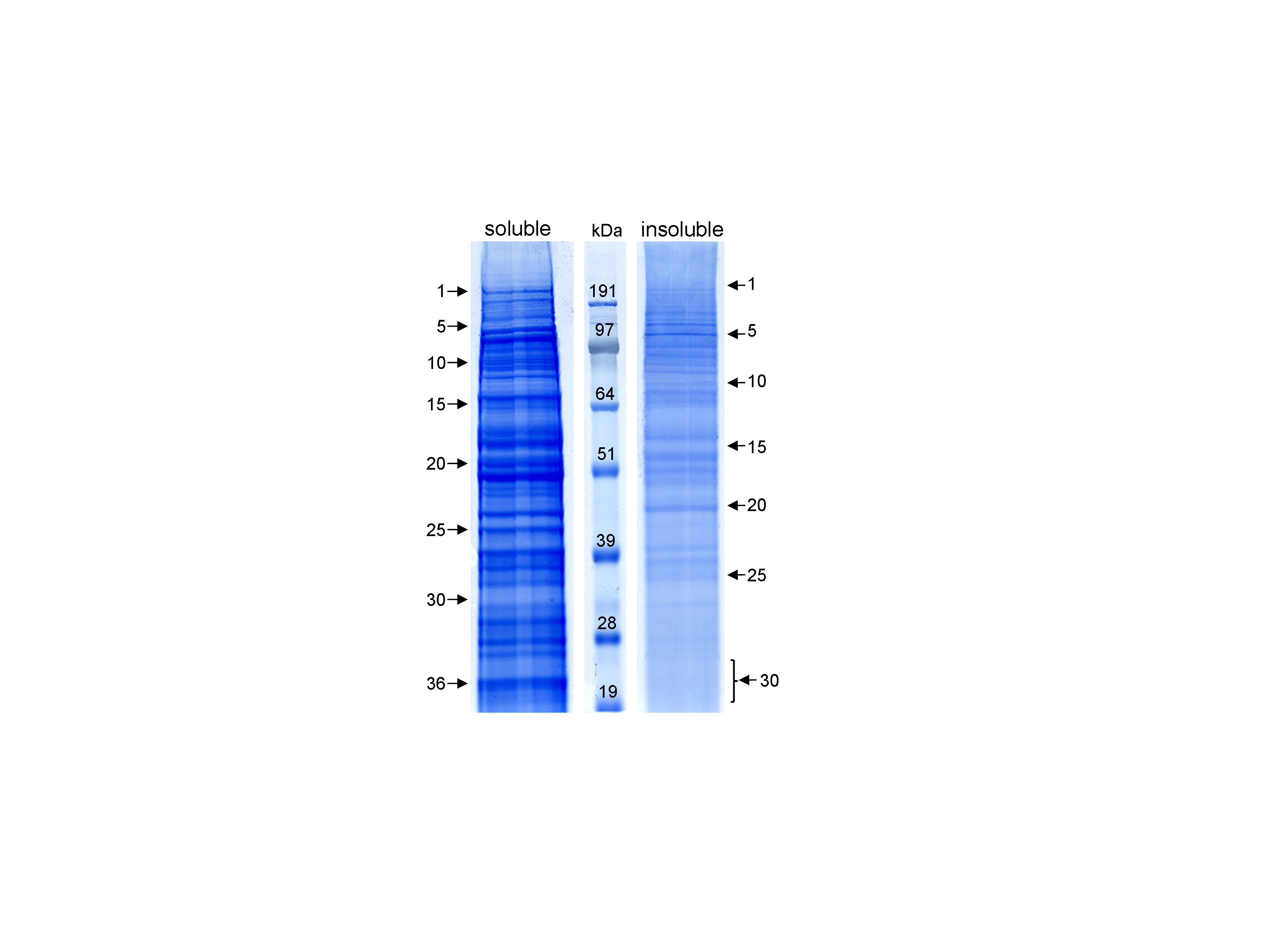

Supplement: Additional file 1: Figure S1 — Electrophoretic profiles of fractionated oocyst/sporozoite proteins of T. gondii. In experiment 3, Tris-soluble and Tris-insoluble protein samples were resolved by SDS-PAGE on a 10% acrylamide gel under reducing conditions and processed for LC-MS/MS analysis. Protein bands were visualized by staining with colloidal Coomassie. The arrows indicate the positions of representative gel slices. [file 1471-2164-14-183-S1.tiff]
